# Osmium–arene complexes with high potency towards *Mycobacterium tuberculosis*

**DOI:** 10.1093/mtomcs/mfab007

**Published:** 2021-03-10

**Authors:** James P C Coverdale, Collette S Guy, Hannah E Bridgewater, Russell J Needham, Elizabeth Fullam, Peter J Sadler

**Affiliations:** Department of Chemistry, University of Warwick, Coventry CV4 7AL, UK; School of Life Sciences, University of Warwick, Coventry CV4 7AL, UK; Department of Chemistry, University of Warwick, Coventry CV4 7AL, UK; Department of Chemistry, University of Warwick, Coventry CV4 7AL, UK; School of Life Sciences, University of Warwick, Coventry CV4 7AL, UK; Department of Chemistry, University of Warwick, Coventry CV4 7AL, UK

**Keywords:** osmium, tuberculosis, mycobacteria, azopyridine, iminopyridine

## Abstract

The treatment of tuberculosis (TB) poses a major challenge as frontline therapeutic agents become increasingly ineffective with the emergence and spread of drug-resistant strains of *Mycobacterium tuberculosis* (*Mtb*). To combat this global health problem, new antitubercular agents with novel modes of action are needed. We have screened a close family of 17 organometallic half-sandwich Os(II) complexes [(arene)Os(phenyl-azo/imino-pyridine)(Cl/I)]^+^Y^–^ containing various arenes (*p*-cymene, biphenyl, or terphenyl), and NMe_2_, F, Cl, or Br phenyl or pyridyl substituents, for activity towards *Mtb* in comparison with normal human lung cells (MRC5). In general, complexes with a monodentate iodido ligand were more potent than chlorido complexes, and the five most potent iodido complexes (MIC 1.25–2.5 µM) have an electron-donating Me_2_N or OH substituent on the phenyl ring. As expected, the counter anion Y (PF_6_^–^, Cl^–^, I^–^) had little effect on the activity. The pattern of potency of the complexes towards *Mtb* is similar to that towards human cells, perhaps because in both cases intracellular thiols are likely to be involved in their activation and their redox mechanism of action. The most active complex against *Mtb* is the *p*-cymene Os(II) NMe_2_-phenyl-azopyridine iodido complex (**2**), a relatively inert complex that also exhibits potent activity towards cancer cells. The uptake of Os from complex **2** by *Mtb* is rapid and peaks after 6 h, with temperature-dependence studies suggesting a major role for active transport.

**Significance to Metallomics**

Antimicrobial resistance is a global health problem. New advances are urgently needed in the discovery of new antibiotics with novel mechanisms of action. Half-sandwich organometallic complexes offer a versatile platform for drug design. We show that with an appropriate choice of the arene, an N,N-chelated ligand, and monodentate ligand, half-sandwich organo–osmium(II) complexes can exhibit potent activity towards *Mycobacterium tuberculosis* (*Mtb*), the leading cause of death from a single infectious agent. The patterns of activity of the 17 azo- and imino-pyridine complexes studied here towards *Mtb* and normal lung cells suggest a common redox mechanism of action involving intracellular thiols.

## Graphical Abstract

**Graphical Abstract:**
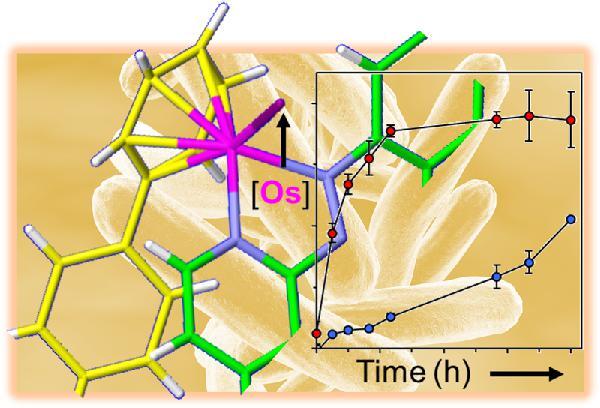
Organo-osmium half-sandwich complexes show high potency towards *Mycobacterium tuberculosis (Mtb).* Overlay of the crystallographic structure of osmium complex **2** and *Mtb* cells with a graph showing cellular accumulation of osmium in *Mtb*.

## Introduction

*Mycobacterium tuberculosis* (*Mtb*), the causative agent of tuberculosis (TB), is a major global health challenge. TB is now the leading cause of death from a single infectious agent, killing more people than HIV and malaria combined.^1^ In 2019, ∼1.4 million people died from this devastating disease and over 10 million new TB cases were reported.^1^ Drug-sensitive TB is curable; however, the complicated 6–9 months treatment regime introduced into the clinic decades ago has gradually become less effective as drug-resistant TB strains have emerged. Treatment of multi-drug resistant (MDR) TB, characterized by resistance to both isoniazid and rifampicin, and extensively drug-resistant (XDR) TB, characterized by resistance to isoniazid, rifampicin, a fluoroquinolone, and one of the injectable second-line drugs, further complicates therapy and often has poor treatment outcomes.^2^ The escalation of MDR TB and XDR TB has, in turn, led to totally drug-resistant strains of TB and in these cases there are no effective therapeutic agents for successful treatment.^3^ Therefore, there is an urgent need to develop new TB drugs that have novel mechanisms of action to reduce the TB burden and tackle this global health crisis.

An analysis of compounds screened for antimicrobial activity by the Community for Open Antimicrobial Drug Discovery has shown that metal complexes have a significantly higher hit rate against critical bacterial and fungal pathogens than organic compounds.^4^ Recent studies on the antimicrobial activity of organometallic complexes (i.e. metal coordination complexes containing metal–carbon bonds) show that they are potentially an encouraging source of novel antibiotics, including complexes of Re, Fe, Ru, Os, Ir, and Au. The C-bound ligands include arenes, cyclopentadienyls, CO, CN^–^, and deprotonated phenyls (often cyclometallated ligands).^5–12^

Half-sandwich ‘piano-stool’ complexes containing a π-bonded neutral arene or negatively charged cyclopentadienyl ligand effectively occupying three coordination sites, and three other donors giving pseudooctahedral geometry (or alternatively described as pseudotetrahedral) are one such class.^13^ Osmium(II)- and Ru(II)-arene complexes, as well as Rh(III)- and Ir(III)-cyclopentadienyl (Cp) complexes have been widely investigated as potential anticancer agents.^14–16^ More recently, the antimicrobial activity of Ir(III), Rh(III), and Ru(II) complexes has been reported, as both 18-electron (6-coordinate) and 16-electron (5-coordinate) half-sandwich complexes.^13,17^ The lipophilicity of the η^6^/η^5^-coordinated arene/Cp ring and the complex can be modulated by extension (e.g. phenylation) of the ring, whilst the monodentate and bidentate ligands allow further structural and electronic control of reactivity, including the incorporation of bio-active moieties and fine-tuning of the redox chemistry of the complex. Both the intact complex and the individual ligands can all play a role in the biological mechanism of action. Such mechanisms can therefore be multi-targeting, perhaps an advantage for overcoming resistance. Surprisingly, although compounds of Os(II) have recently found recognition as potential anticancer agents,^18–20^ their application to the treatment of tuberculosis has not yet been explored. Os(II) arene azopyridine and iminopyridine complexes have been shown to be highly potent towards cancer cells, with some selectivity for cancer cells versus normal human cells.^21–23^

In this work, we investigate the activity of a family of structurally related osmium–arene complexes against *Mtb* and normal lung cells. We also study the time dependence and temperature dependence of intracellular osmium accumulation in *Mtb* for one of the most potent complexes.

## Results and discussion

### Antibacterial potency

**Fig. 1:**
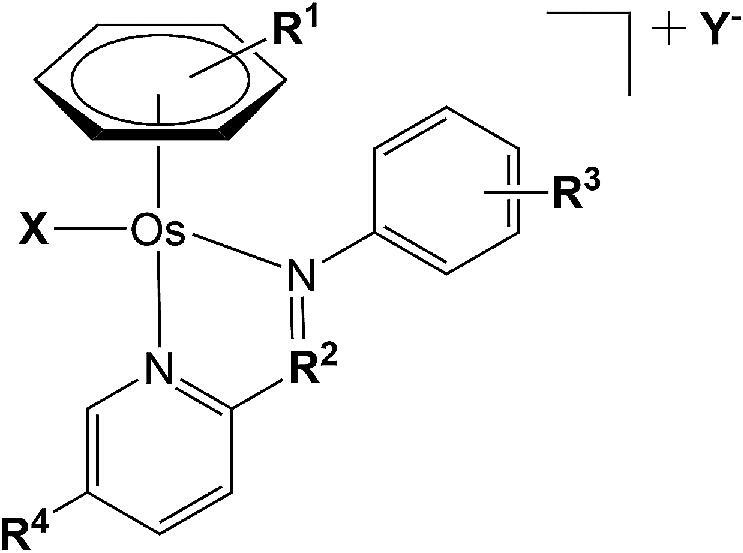
Generic structure of Os(II) arene ‘piano-stool’ complexes studied in this work.

Minimum inhibitory concentrations (MICs) against *Mtb* were determined for a series of 17 structurally related ‘piano-stool’ osmium(II) *p*-cymene (*para*-cymene) or biphenyl arene, azo- and imino-pyridine complexes containing either chloride or iodide as a monodentate ligand (Fig. 1). The MIC testing was carried out in Middlebrook 7H9 broth supplemented with 0.2% glycerol, 0.05% Tween 80, and 10% albumin–dextrose–catalase (ADC).

Previous work has shown that iodido complexes in this series are usually relatively inert, e.g. towards aquation, in comparison with chlorido complexes. It is notable that these small changes in the ligands have a significant effect on the activities, ranging across two orders of magnitude (1.25–125 µM, Table 1).

**Table 1.** Biological activities of osmium arene complexes tested against *Mtb* (H37Rv) and normal human MRC5 lung fibroblasts
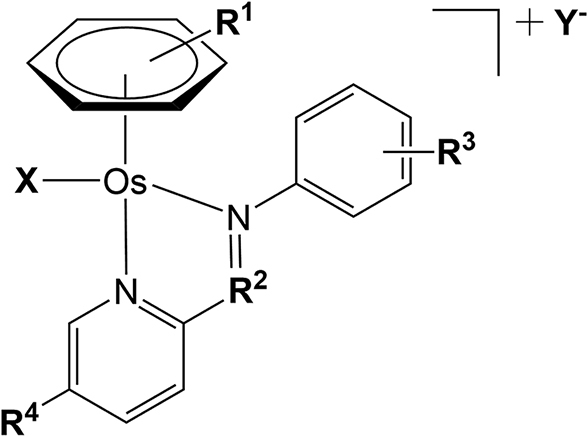

	R^1^	R^2^	R^3^	R^4^	X	Y^–^	MIC/µM^a^*Mtb* H37Rv	IC_50_/µM^b^ Human MRC5
**1**	*p*-cym	N	3-NMe_2_	H	Cl	PF_6_	5	7.1 ± 0.2
**2**	*p*-cym	N	3-NMe_2_	H	I	PF_6_	1.25	0.89 ± 0.01
**3**	biphenyl	N	3-NMe_2_	H	Cl	PF_6_	31.25	16.9 ± 0.1
**4**	biphenyl	N	3-NMe_2_	H	I	PF_6_	2.5	0.42 ± 0.03
**5**	*p*-cym	CH	3-NMe_2_	H	Cl	PF_6_	31.25	7.6 ± 0.9
**6**	*p*-cym	CH	3-NMe_2_	H	I	PF_6_	7.81	13 ± 2
**7**	*p*-cym	N	3-NMe_2_	H	I	Cl	1.25	0.68 ± 0.03
**8**	*p*-cym	N	3-NMe_2_	H	I	I	1.25	0.42 ± 0.01
**9**	*p*-cym	N	H	H	Cl	PF_6_	15.6	15 ± 2
**10**	*p*-cym	N	H	H	I	PF_6_	5	0.79 ± 0.06
**11**	*p*-cym	N	H	5-Br	Cl	PF_6_	125	18 ± 1
**12**	*p*-cym	N	H	5-F	I	PF_6_	15.6	9.1 ± 0.6
**13**	biphenyl	N	H	5-F	I	PF_6_	62.5	5.2 ± 0.7
**14**	*p*-cym	N	H	5-Cl	I	PF_6_	62.5	16 ± 2
**15**	biphenyl	CH	3-OH	H	I	PF_6_	5	13.5 ± 0.9
**16**	*p*-cym	N	3-OH	Br	Cl	PF_6_	10	0.08 ± 0.01
**17**	*p*-cym	N	3-OH	Br	I	PF_6_	2.5	0.62 ± 0.01
**Rifampicin**	N/A	N/A	N/A	N/A	N/A	N/A	0.38–0.76	n.d.
**Cisplatin**	N/A	N/A	N/A	N/A	N/A	N/A	n.d.	13.5 ± 0.9

^a^MIC determined using resazurin, after 7-day exposure to the Os(II) complex (results represent three independent biological repeats); the medium used was Middlebrook 7H9 broth supplemented with 0.2% glycerol, 0.05% Tween 80, and 10% ADC. MBC values were obtained for **1** (12.5 µM), **2** (6.25 µM), and **4** (6.25 µM).

^b^IC_50_ determined using the SRB assay after 24 h drug exposure and 72 h recovery time in drug-free medium (results represent three independent biological repeats). N/A = not applicable; n.d. = not determined.

For this family of osmium complexes, biphenyl (R^1^) complexes often exhibit higher activity towards cancer cell lines than their *p-*cymene analogues.^24–26^ However, osmium–biphenyl (R^1^) complexes appear less potent towards *Mtb* than their *p*-cymene analogues, e.g. MIC of 31.25 µM for **3** compared with 5 µM for **1**. The R^1^ arene substituent can increase lipophilicity and promote uptake of the complex into cells, which is particularly important for import into *Mtb* as it has a complicated lipid-rich cell envelope that is highly impenetrable to most antibiotics.^27^

Complexes with an iodido monodentate ligand (X) were between 2 and 12.5× more active against *Mtb* than their chlorido counterparts, c.f. **1** vs **2**, **3** vs **4**, **5** vs **6**, **9** vs **10**, and **16** vs **17**, a trend commonly observed for mammalian cancer cell lines studies.^24,26^ Chemical and radiotracer studies using ^131^I (β^−^/γ emitter, t_1/2_ 8.02 days) suggest that the iodido azopyridine complexes, which are relatively inert towards ligand exchange reactions, are activated inside human carcinoma cells,^28^ through attack on the azo bond by the thiol of glutathione (GSH), which is abundant (millimolar concentrations) in cells*.* Initially, this leads to release of the iodide ligand and formation of the more reactive chlorido and hydroxido adducts in cells. Then, thiolato (GS) and sulfenato (GSO) adducts can form, which can react with hydrogen peroxide, generating reactive oxygen species (ROS) inside cells.^28,29^ Thiols may also be involved in the intracellular activation of these potent osmium–arene complexes in *Mtb* cells. Whilst *Mtb* lacks the glutathione system, there are a number of thiols present at similar levels to those of GSH in mammalian cells: ergothioneine (ERG), mycothiol (MSH), and γ-glutamylcysteine (GGC).^30,31^ They probably fulfil similar roles: maintaining the redox balance and counteracting ROS, protecting against oxidative stress (ROS), and are likely to interact with the osmium complexes as GSH does in mammalian cells.^32^

Introduction of an R^3^ = NMe_2_ substituent onto the phenyl ring of the azopyridine bidentate ligand (complexes **1** and **2**) enhances activity against *Mtb*, compared with unsubstituted (R^3^ = H) counterparts, complexes **9** and **10**, respectively. This group is known to enhance potency and selectivity in human cell lines,^22,23,28^ and also appears to contribute to the overall antimycobacterial activity. Imino complexes **5** and **6** were less potent towards *Mtb* than their azo complex analogues **1** and **2**, respectively, suggesting that the nature of the azo bond is important. It seems likely that redox reactions involving the azo bond may be involved not only in the mechanism of cytotoxicity towards human cancer cells but also towards *Mtb*.^33^

Generally, introduction of halide (electron-withdrawing) moieties at the R^4^ position was detrimental to both mammalian and mycobacterial potencies (compounds **11–14**), with potency inversely correlated with the size of the halide substituent: **11** (R^4^ = Br, least active) < **14** (R^4^ = Cl) < **12** (R^4^ = *F*, most active) (Table 1). Conversely, and although directly comparable complexes were not available, the introduction of Br at the R^4^ position in R^3^ = 3-OH complexes (**16** and **17**) did not appear to affect significantly the potency in either mammalian or mycobacterial cells. The apparent inertness of this site towards substitution reactions suggests that it may be suitable for further investigation (e.g. polymer conjugation as part of a future drug-delivery strategy).

Complex **2** [Os(AzPy-NMe_2_)I(*p*-cymene)]PF_6_ (AzPy: 2-phenylazopyridine) has previously shown promise as a candidate anticancer drug, with selectivity for cancer cells over healthy cells.^34^ In our current work, this compound also possesses high antitubercular activity, with an MIC potency of 1.25 µM, which is comparable with MIC values of ∼1–9 µM for current clinical TB drugs isoniazid (∼1 µM), ethambutol (∼5 µM), capreomycin (*∼*3 µM), and streptomycin (∼9 µM). However, complex **2** has a lack of selectivity towards normal human cells compared with the bacteria (Table 1). We have recently reported the procedure for exchange of the counter anion in these complexes, using anion exchange resin and column chromatography.^35^ As might be expected, since the complexes are salts, variation in the counter anion for complex **2** from X^–^ = PF_6_^–^, to Cl^–^ (complex **7**), or I^–^ (complex **8**) had little effect on the antimicrobial activity of the compound (1.25 µM), although this change in halide counter ion has a significant effect on the aqueous solubility of the complexes (**7** >> **8** > **2**).

The minimal bactericidal concentrations (MBCs) were determined for compound **1** (12.5 µM), compound **2** (6.25 µM), and compound **4** (6.25 µM). Compared with the determined MIC values (Table 1), there is good correlation with the MBCs for compounds **1** and **4**, indicating that these compounds are bactericidal against actively growing *Mtb*, whereas compound **2** displayed an MBC 5× greater than the corresponding MIC value.

**Fig. 2:**
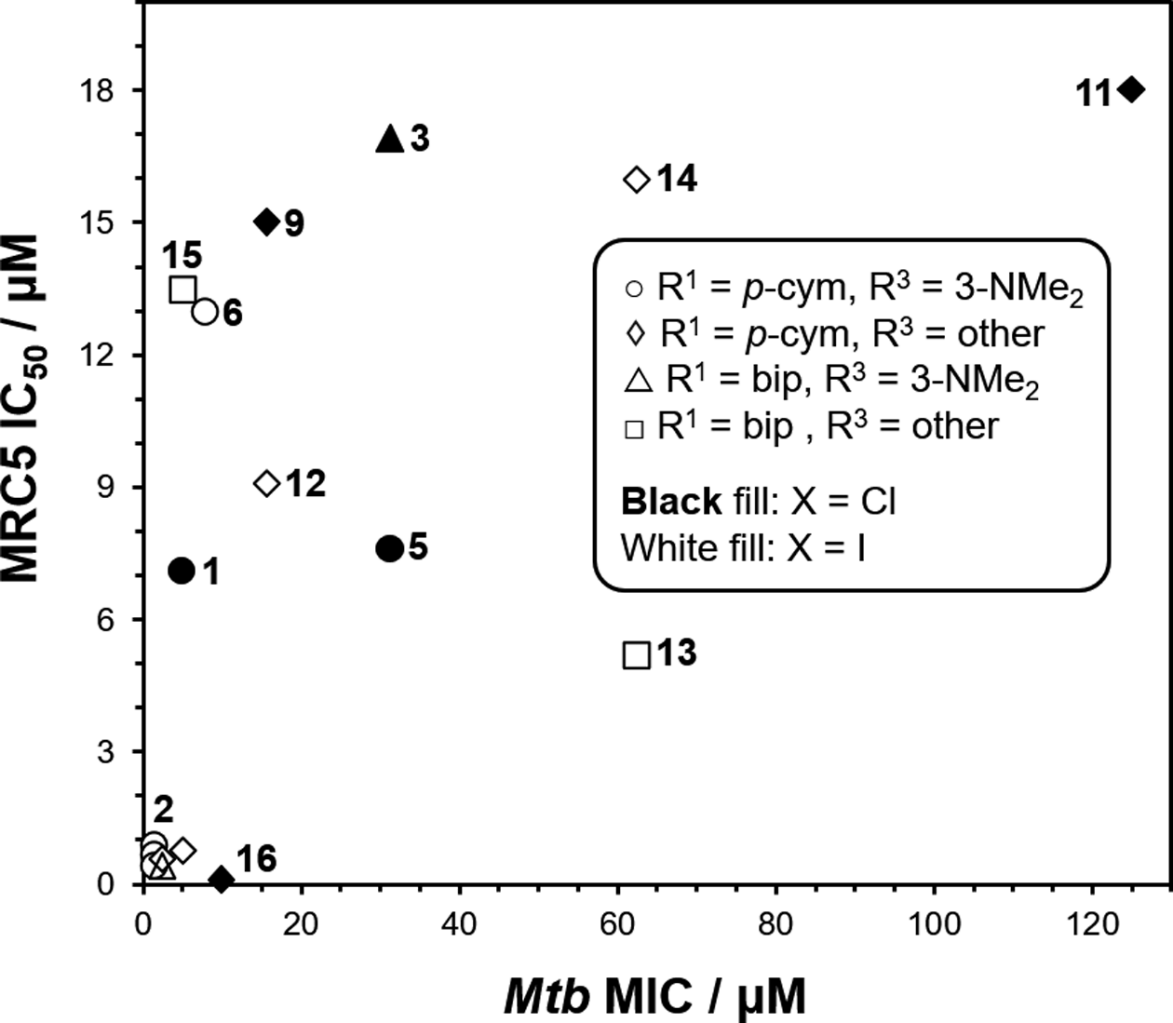
Comparison of the cytotoxicity of complexes **1–17** towards *Mtb* (MIC µM values) and normal MRC5 human lung fibroblasts (IC_50_ µM values). In general, high activity against *Mtb* is paralleled by high activity towards the human cells, with low selectivity for *Mtb* cells observed. The results represent three biological repeats. Points are labelled with complex numbers according to Table 1, except for highly active complexes **4**, **7**, **8**, **10**, and **17**, which are clustered near the origin.

Comparable activity trends in MIC and IC_50_ (half-maximal inhibitory concentrations) are observed for *Mtb* and MRC5 normal human lung cell lines (Table 1; Fig. 2). Iodido complexes are more active than their chlorido counterparts, and azo ligands impart greater antiproliferative activity than their imino analogues. In general, there is little selectivity for *Mtb* versus the human cells MRC5 lung fibroblasts (Table 1; Fig. 2). However, as shown in Fig. 2, complexes **6**, **9**, and **15** do show higher relative potency for *Mtb* compared with the MRC5 cells, suggesting that these complexes could act as leads for further optimization.

### Accumulation of osmium in cells

**Fig. 3:**
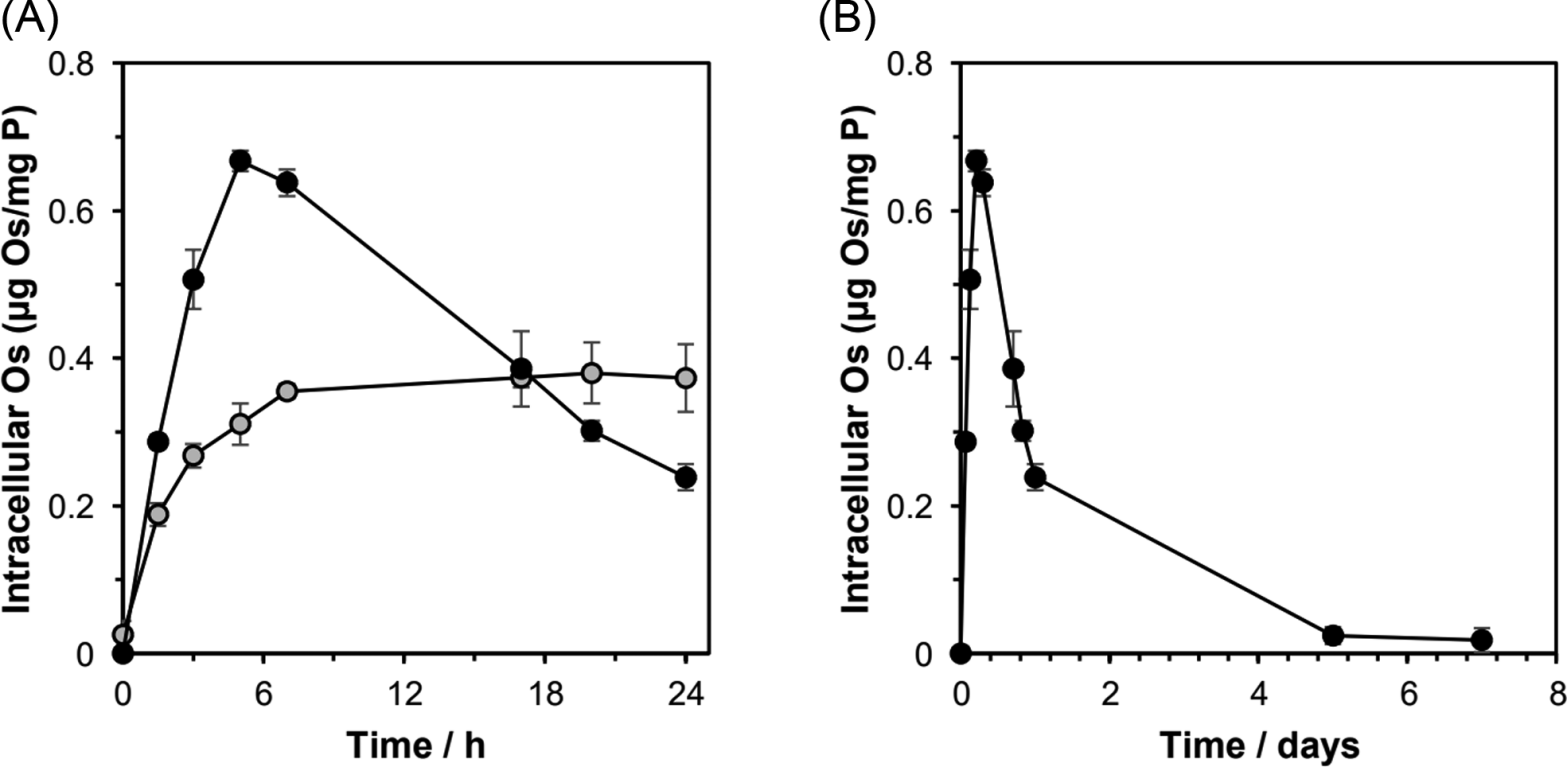
Time-dependent accumulation of Os from complex **2** by *Mtb* H37Rv cells (determined by ICP-MS), normalized to phosphorus content. Cells were treated using 0.5× MIC (0.625 µM) concentrations of complex **2** and incubated at 310 K. (A) During incubation time, not shaking the culture (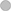) was found to decrease the rate of osmium accumulation over the first 6 h of incubation, relative to the comparative experiment with shaking (●); (B) with shaking (●), osmium accumulation reaches a peak after 6 h of exposure; however, after 5 days, the intracellular osmium concentration is negligible. Full numerical data can be found in Table S1. Error bars represent ± SD.

*Mtb* cells were exposed to complex **2** for 24 h at a concentration of 0.625 µM (0.5 × MIC), with time-dependent sampling from the culture either with or without shaking. Shaking was found to affect significantly the Os accumulation profile (Fig. 3A), increasing the initial rate of Os accumulation, leading to peak accumulation after 6 h. In contrast, *Mtb* cells that were cultured without shaking accumulated Os at a much slower rate, with an accumulation maximum being reached after ∼17 h.

For *Mtb* cells incubated with shaking, the intracellular concentration of Os decreased after 6 h, and after 5 days was negligible, suggesting an active contribution from efflux mechanisms, irrespective of the extracellular Os concentration gradient (Fig. 3B). *Mtb* cellular osmium accumulation is a result of two processes: influx and efflux, and these data suggest that, after 6 h, either: (i) the rate osmium of influx must decrease by an unknown mechanism (to a rate less than the rate of efflux), or (ii) the rate of osmium efflux must increase (and surpass the rate of influx). Similar accumulation profiles have been observed and modelled for mammalian cancer cells.^36^ These models demonstrated that, in mammalian cells, the reduction in intracellular Os is a consequence of reduced osmium influx, rather than increased efflux, after reaching a critical intracellular concentration threshold.^36^

**Fig. 4:**
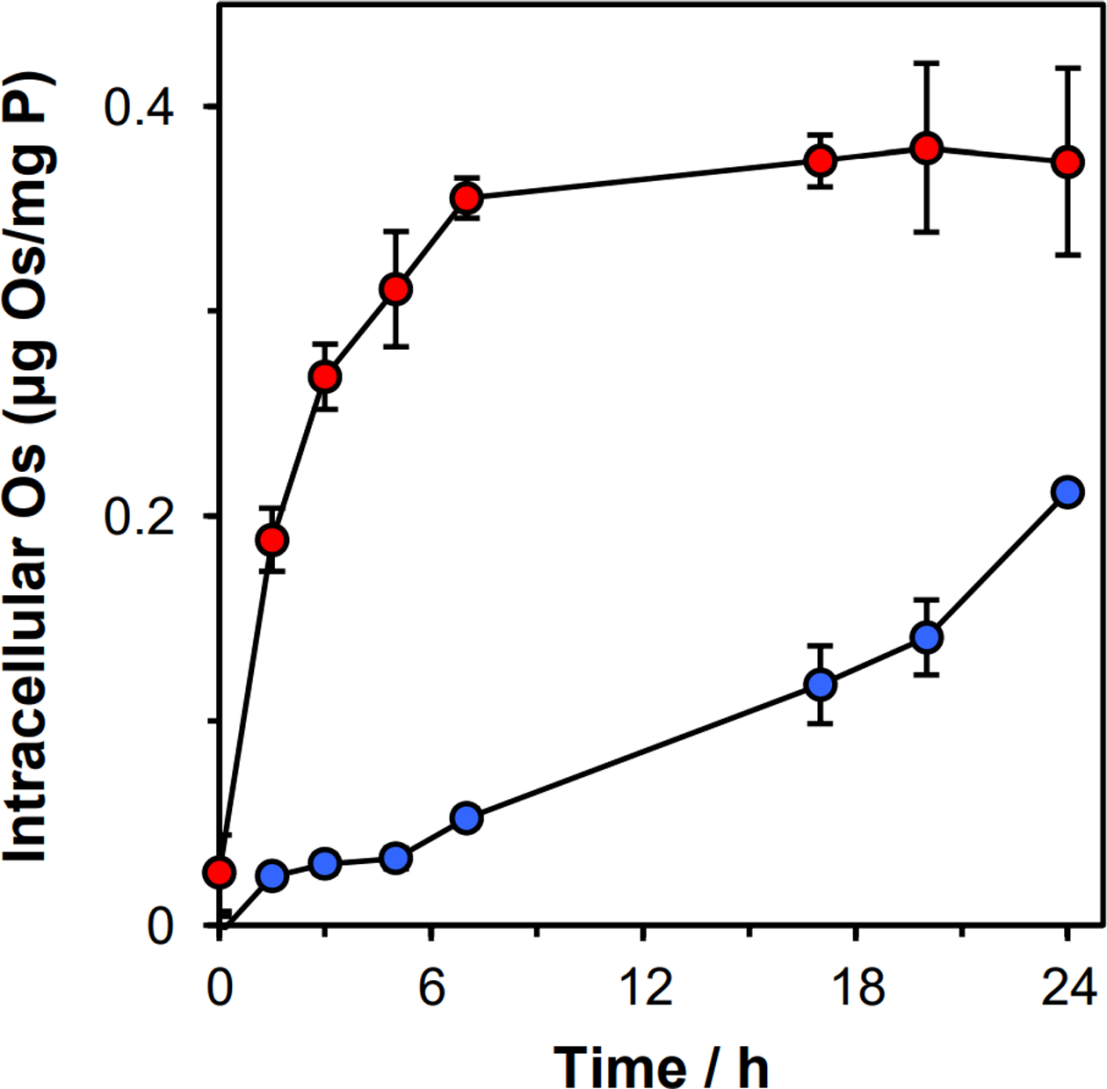
Temperature-dependent accumulation of Os (ICP-MS) by *Mtb*, normalized to phosphorus content (ICP-OES). Cells were treated using 0.5× MIC (0.625 µM) of complex **2** and incubated at 277 K without shaking (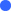) and are shown compared with data obtained at 310 K without shaking (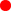). Full numerical data can be found in Table S2. Error bars are ±SD.

The temperature dependence of osmium accumulation was next investigated by incubating *Mtb* at 277 K with complex **2** in a time-dependent manner in static cultures. These data demonstrate that Os accumulation depends on contributions from passive and active transport mechanisms. Active transport requires energy; therefore, at lower temperatures active transport would be diminished. Osmium accumulation at 277 K suggests passive diffusion of the complex across the lipid envelope (Fig. 4). Previous studies have demonstrated that the intracellular Os accumulation also decreased at lower temperature in mammalian cells: by 4-fold at 296 K, and by 31-fold at 277 K, compared with the intracellular concentration determined at 310 K.^36^ Since osmium from complex **2** was still accumulated at low temperature (in both *Mtb* and mammalian cells), this suggests that its uptake is partially passive and not entirely energy-dependent.

## Conclusions

The well-known compound OsO_4,_ a strong oxidant used as a stain for cells, and intriguingly for treatment of synovitis of the knee,^37^ contains osmium in oxidation state +8, in contrast to the Os(II) complexes used in the present work. The chemical and biological properties of osmium complexes depend not only on the oxidation state of osmium but also on the nature of the coordinated ligands and the geometry of the complex. This is clear from the anti-*Mtb* activity of complexes **1–17** that ranges from 1.25 to 125 µM. Moreover, these complexes form a close family, with the same half-sandwich structures and only small variations in the ligands. For changes in the N,N-chelated ligand, the activity increases for azo > imino, and for the monodentate ligand I > Cl. The 4–6× increase in activity conferred by the presence of the NMe_2_ group on the phenyl ring of the N,N-chelated ligand is also notable. This activity pattern is paralleled in activity towards human cancer cells.^24–26^ Although complex **15** shows selectivity for *Mtb* versus normal human cells by a factor of 3 (Fig. 2), the selectivity needs to be improved for clinical development. However, selectivity for microbes versus human cells in culture, although a desirable initial goal, may not translate to selectivity in the body where additional metabolic processes can occur in various organs and tissues (depending on the route of administration). Selectivity might be improved by conjugation of complexes to targeting vectors for *Mtb* to the ligands, or by formulation in nanoparticles that introduce selective delivery.

Compared with cancer cells, little work has been done so far on understanding intracellular reactions of antimicrobial metallodrugs. In the present case, it seems likely that inert organo–osmium complexes are activated in *Mtb* by reaction with intracellular thiols, as they are in mammalian cells,^28^ generating ROS.^38^ γ-Glutamylcysteine, a precursor to GSH in mammalian cells, in *Mtb* is likely to react similarly to GSH, and it will be interesting to compare with ergothioneine and mycothiol.

Current widely used transition metal drugs in clinical use include the platinum anticancer drug cisplatin and antimicrobial silver compounds such as silver sulfadiazine.^39^ The attraction of such metal compounds as drugs relates partly to their novel mechanisms of action compared with organic drugs.^4^ Organo-osmium complexes are therefore worthy of further investigation.

## Experimental Materials

Osmium(II) azopyridine and osmium(II) iminopyridine complexes have been previously reported and fully characterized. Changes in the counter anions (e.g. conversion of PF_6_^–^ salt **2** to Cl^–^ salt **7**) were carried out by ion-exchange chromatography as described recently.^35^

DMEM culture medium was purchased from Scientific Laboratory supplies. Foetal calf serum (batch 70428) was purchased from Lab Tech International. Osmium trichloride was received from Heraeus GmbH. Plasticware for biological experiments was purchased from Greiner Bio-One. Ultrapure nitric acid (72% v/v) was freshly distilled before use. All water used in experiments was Type I Milli-Q (doubly deionized) water. ICP standards for Os were purchased from Inorganic Ventures. All other reagents were purchased from Sigma Aldrich (Merck) and used as received, unless indicated otherwise.

## Synthesis of L1

*p*-Benzoquinone (316.0 mg, 2.93 mmol) was dissolved in deionized water (50 mL) and perchloric acid (70% v/v, 2.6 mL) was added. A solution of 5-bromo-2-hydrazinopyridine (500.0 mg, 2.66 mmol) in MeOH (10 mL) was added dropwise to the stirring mixture. The mixture turned red-brown and was stirred for 18 h at ambient temperature. The pH was neutralized with the dropwise addition of aqueous sodium hydroxide (6 M). The product was extracted with ethyl acetate (3 × 50 mL) and washed with water (3 × 50 mL). The mixture was concentrated under reduced pressure and placed in a freezer (253 K) overnight. The resulting brown precipitate was collected via vacuum filtration and washed with ice-cold EtOH (2 × 1 mL) and Et_2_O (2 × 5 mL). Yield: 495.5 mg (67%). ^1^H NMR (400 MHz, CD_3_OD): *δ* 8.73 (d, 1H, *J* = 2.4 Hz), 8.19 (dd, 1H, *J* = 8.6, 2.4 Hz), 7.93–7.92 (m, 2H), 7.76 (d, 1H, *J* = 8.6 Hz), 6.96–6.94 (m, 2H). ESI-MS calculated for C_11_H_9_BrN_3_O^+^: m/z 278.0. Found: 277.9. CHN analysis: Found: C, 47.28%; H, 2.80%; N, 14.82%. Calculated for C_11_H_8_BrN_3_O: C, 47.51%; H, 2.90%; N, 15.11%.

## Synthesis of complex 16

[Os(η^6^-*p*-cym)Cl_2_]_2_ (50.0 mg, 63.2 µmol) was dissolved in EtOH (10 mL), and a solution of 5-Br-AzPy-OH (**L1**, 36.9 mg, 132.8 µmol) in EtOH (5 mL) was added dropwise. The mixture was stirred for 18 h at ambient temperature and then filtered through glass microfibre to remove a black precipitate, and NH_4_PF_6_ (103.1 mg, 0.63 mmol) was added. The mixture was concentrated under reduced pressure to ∼3 mL and placed in a freezer (253 K) overnight. A dark crystalline precipitate was collected via vacuum filtration and washed with ice-cold EtOH (2 × 1 mL) and Et_2_O (2 × 5 mL) and dried overnight in a vacuum desiccator. Yield: 89.6 mg (91%). ^1^H NMR (400 MHz, (CD_3_)_2_CO): *δ* 9.66 (d, 1H, *J* = 2.1 Hz), 8.75 (d, 1H, *J* = 8.7 Hz), 8.56 (dd, 1H, *J *= 8.7, 2.1 Hz), 8.15–8.11 (m, 2H), 7.15–7.11 (m, 2H), 6.82–6.81 (m, 1H), 6.53–6.52 (m, 1H), 6.40–6.35 (m, 2H), 2.47 (sept., 1H, *J* = 6.9 Hz), 2.43 (s, 3H), 0.97 (d, 3H, *J* = 6.9 Hz), and 0.91 (d, 3H, *J* = 6.9 Hz). ESI-MS calculated for C_21_H_22_BrClN_3_OOs^+^: m/z 638.0. Found: 637.9. CHN analysis: Found: C, 31.87%; H, 2.70%; N, 5.16%. Calculated for C_21_H_22_BrClF_6_N_3_OOsP: C, 32.21%; H, 2.83%; N, 5.37%. HPLC purity 98.3%.

## Synthesis of complex 17

[Os(η^6^-*p*-cymene)I_2_]_2_ (100.0 mg, 86.5 µmol) was dissolved in EtOH (10 mL), and a solution of 5-Br-AzPy-OH (**L1**, 50.5 mg, 181.6 µmol) in EtOH (5 mL) was added dropwise. The mixture was stirred for 18 h at ambient temperature and then filtered through glass microfibre to remove a black precipitate, and NH_4_PF_6_ (140.9 mg, 0.87 mmol) was added. The mixture was concentrated under reduced pressure to ∼3 mL and placed in a freezer (253 K) overnight. A dark crystalline precipitate was collected via vacuum filtration and washed with ice-cold EtOH (2 × 1 mL) and Et_2_O (2 × 5 mL) and dried overnight in a vacuum desiccator. Yield: 76.7 mg (51%). ^1^H NMR (400 MHz, CD_3_OD): *δ* 9.10–9.09 (m, 1H), 8.12–8.08 (m, 2H), 8.02–8.01 (m, 2H), 6.43–6.39 (m, 2H), 7.15–7.11 (m, 2H), 6.20–6.19 (m, 1H), 6.07–6.06 (m, 1H), 6.03–6.02 (m, 1H), 5.97–5.96 (m, 1H), 2.73 (s, 3H), 2.39 (sept., 1H, *J* = 6.9 Hz), 0.97 (d, 3H, *J* = 6.9 Hz), and 0.89 (d, 3H, *J* = 6.9 Hz). ESI-MS calculated for C_21_H_22_BrIN_3_OOs^+^: m/z 730.0. Found: 729.8. CHN analysis: Found: C, 28.62%; H, 2.63%; N, 4.73%. Calculated for C_21_H_22_BrF_6_IN_3_OOsP: C, 28.85%; H, 2.54%; N, 4.81%. HPLC purity 99.7%.

## Bacterial strains and culture conditions

*Mtb* H37Rv was routinely grown at 37°C in Middlebrook 7H9 broth (BD Difco, Oxford, UK) supplemented with 0.2% glycerol, 0.05% Tween 80, and 10% ADC or on Middlebrook 7H10 plates supplemented with 10% glycerol and 10% oleic acid–albumin–dextrose–catalase (OADC). *Mtb* was cultured within a containment level 3 (CL3) facility. *Mtb* samples were removed from the CL3 containment only after validation that *Mtb* was killed following nitric acid treatment.

## Determination of MICs against *Mtb*

The MIC of all compounds was determined using the resazurin microtitre assay, as described previously.^40^ Compounds were dissolved in dimethylsulfoxide (DMSO) and then diluted so that the final concentration in the media did not exceed 2% v/v. *Mtb* H37Rv was grown to mid-log phase [optical density at 600 nm (OD_600_) of 0.6] and the inoculum was standardized to 1 × 10^6^ colony-forming units (CFUs)/mL before addition to a prepared 96-well flat bottom microtitre plate with 2-fold serial dilutions of each compound in media. Control experiments in which either no drug, or rifampicin as an antibiotic control were also included on each microtitre plate. The plates were incubated for 7 days at 310 K before the addition of 25 µL resazurin [one tablet of resazurin (VWR) dissolved in 30 mL of sterile phosphate buffered saline (PBS). Following a further incubation for 24 h at 310 K, the plates were assessed for colour development. The MIC values were determined as the lowest concentration of drug that prevented the turnover of resazurin from blue (no bacterial growth) to resorufin, pink (bacterial growth). The results represent three independent biological repeats.

## Determination of minimum bactericidal concentrations against *Mtb*

The minimum bactericidal activity (MBC) was determined by setting up a microtitre plate as performed for the MIC determination. Instead of adding resazurin, each well from the microtitre plate was plated on to solid 7H10 medium and the CFUs were determined after incubation at 37°C. The lowest concentration at which no CFUs were counted was taken as the MBC. The MBCs were carried out with three independent experimental repeats.

## Os uptake experiments in *Mtb*

*Mtb* H37Rv was grown to an OD_600_ of 0.5 and then either compound **2** (final concentration of 625 nM) or water was added. The cultures were incubated at either 277 or 310 K over 24 h or 7 days. At each time point, *Mtb* culture (1.5 mL) was removed, centrifuged (10 min, 15 871 × *g*), the supernatant removed, and the pellet washed three times with Tris-buffered-saline containing 0.05% Tween 80 (TBST). The pellet was resuspended in 72% v/v nitric acid (200 µL), heated to 353 K for 16 h, cooled to room temperature, and diluted with Milli-Q water containing 100 mg L^–1^ ascorbic acid and 10 mM thiourea (3.8 mL). The results represent three independent experimental repeats.

## Human cell line maintenance

MRC5 (human foetal lung fibroblast) cell lines were obtained from Public Health England (mycoplasma-free status confirmed at six-monthly intervals by internal and external screening). They were thawed upon receipt and the supernatant removed after centrifugation (1000 rpm, 5 min). Cells were re-suspended in DMEM culture medium (supplemented with 10% v/v FCS, 1% penicillin/streptomycin, and 1% l-glutamine) and grown as adherent monolayers at 310 K in a 5% CO_2_ humidified atmosphere. Cells were passaged using trypsin/EDTA after reaching ∼90% confluence. Trypsin activity was quenched by addition of excess medium, and the single-cell suspension passaged twice weekly. MRC5 fibroblasts were discarded after five passages.

## Determination of antiproliferative activities against human cell line

Briefly, 5 × 10^3^ cells (MRC5) were seeded in a 96-well plate using 0.15 mL of culture medium. After 48 h pre-incubation at 310 K, the supernatant was removed and cells exposed to six concentrations (0–100 µM) of osmium complex, for which exact concentrations were determined using ICP-OES (calibration standards for Os freshly prepared in 3.6% v/v nitric acid containing 100 mg L^–1^ ascorbic acid and 10 mM thiourea). DMSO was used to aid solubility of Os complexes, but never exceeded 0.5% v/v in the final culture medium. Cells were exposed to Os complexes for 24 h, and then washed with PBS and allowed 72 h recovery time in drug-free medium. Cell viability was determined using the sulforhodamine B (SRB) assay. Cells were fixed using 50 µL of 50% TCA, incubated at 277 K for 1 h, and then stained using SRB dye (0.4% dye in 1% acetic acid). After 30 min, excess dye was removed by acetic acid washing, and then the stain liberated using Tris base (10 mM, pH 10.5). Absorbance was measured using a Thermo Scientific Multiskan FC microplate reader fitted with a 492-nm filter. Data were processed using Microsoft Excel and are reported as percentage survival relative to the untreated control. Sigmoidal dose/response curves were fitted using Origin 2016 to determine IC_50_. Data were acquired as duplicate of triplicate experiments, with a positive control of cisplatin (CDDP).

## Quantification of cellular Os and P

Cell pellets were obtained and subjected to digestion using 200 µL of 72% v/v nitric acid. After 24 h incubation at 353 K, samples were diluted using 3800 µL Milli-Q water containing 100 mg L^–1^ ascorbic acid and 10 mM thiourea to stabilize Os in nitric acid solution. Samples were analysed using an Agilent Technologies 7900 Series ICP-MS (inductively coupled plasma-mass spectrometry) in He gas mode. Calibration standards (0–1000 ppb) for ^189^Os and ^31^P were freshly prepared in 3.6% v/v nitric acid containing 100 mg L^–1^ ascorbic acid and 10 mM thiourea, and all samples analysed using an internal standard of ^166^Er. Osmium accumulation was normalized to phosphorus content (µg Os/mg P) and standard deviations are reported.

## Supplementary Material

mfab007_Supplemental_File

## Data Availability

The data underlying this article are available in the Warwick Research Archive Portal (WRAP) at http://wrap.warwick.ac.uk/148351 and can be accessed as Dataset #148351.
