# Supplementary material for: Osmium–arene complexes with high potency towards Mycobacterium tuberculosis
Source: Metallomics. 2021 Mar 10;13(4):mfab007. doi: 10.1093/mtomcs/mfab007 (PMC8026400; doi:10.1093/mtomcs/mfab007)
Supplement: mfab007_Supplemental_File [file mfab007_supplemental_file.docx]

**Electronic Supplementary information (ESI)**

**Osmium-arene complexes with high potency towards *Mycobacterium tuberculosis***

James P. C. Coverdale,^[1][a]^ Collette S. Guy,^[1][b]^ Hannah E. Bridgewater,^[a]^ Russell J. Needham,^[a]^ Elizabeth Fullam,^[b]*^ and Peter J. Sadler^[a]*^

^[a]^ Department of Chemistry, University of Warwick, UK, CV4 7AL, ^[b]^ School of Life Sciences, University of Warwick, UK, CV4 7AL.

* E-mail: [P.J.Sadler@warwick.ac.uk](mailto:P.J.Sadler@warwick.ac.uk); e.fullam@warwick.ac.uk

**Table S1.** Full numerical data for time-dependent accumulation of Os in *Mtb* treated with complex **2**

**Table S2.** Full numerical data for temperature-dependent accumulation of Os in *Mtb* treated with complex **2**

**Table S1.** Full numerical data for time-dependent accumulation of Os in *Mtb* treated with complex **2** 0.5× MIC) and incubated at 310 K (shaking included during incubation time). Os accumulation determined by ICP-MS and normalised to P content.

| **Time-dependent Os accumulation (µg Os / mg P)** | |
| --- | --- |
| **Time / h (days)** | **310 K** |
| 0 | 0 ± 0.009 |
| 1.5 | 0.287 ± 0.009 |
| 3 | 0.51 ± 0.04 |
| 5 | 0.67 ± 0.01 |
| 7 | 0.64 ± 0.02 |
| 17 | 0.39 ± 0.05 |
| 20 | 0.30 ± 0.01 |
| 24 (1) | 0.24 ± 0.02 |
| 120 (5) | 0.02 ± 0.01 |
| 168 (7) | 0.02 ± 0.02 |

**Table S2.** Full numerical data for temperature-dependent accumulation of Os in *Mtb* treated with complex **2** (0.5× MIC) and incubated at either 277 K or 310 K (no shaking during incubation time). Os accumulation determined by ICP-MS and normalised to P content.

| **Temperature-dependent Os accumulation (µg Os / mg P)** | | |
| --- | --- | --- |
| **Time / h** | **277 K** | **310 K** |
| 0 | 0.000 ± 0.009 | 0.02 ± 0.02 |
| 1.5 | 0.024 ± 0.001 | 0.19 ± 0.02 |
| 3 | 0.030 ± 0.005 | 0.27 ± 0.02 |
| 5 | 0.033 ± 0.005 | 0.31 ± 0.03 |
| 7 | 0.052 ± 0.005 | 0.36 ± 0.01 |
| 17 | 0.12 ± 0.02 | 0.37 ± 0.01 |
| 20 | 0.14 ± 0.02 | 0.38 ± 0.04 |
| 24 | 0.211 ± 0.004 | 0.37 ± 0.05 |
